# Guideline for Analysis and Prevention of Contamination Catalysis

**DOI:** 10.1002/anie.202424425

**Published:** 2025-04-30

**Authors:** János Daru, Zsombor Gonda, Zoltán May, Zoltán Novák, Gergely L. Tolnai

**Affiliations:** ^1^ Institute of Chemistry Eötvös Loránd University Pázmány Péter stny. 1/A Budapest H‐1117 Hungary; ^2^ Research Centre for Natural Sciences HUN‐REN Magyar Tudósok körútja 2 Budapest H‐1117 Hungary

**Keywords:** Catalysis, Contamination, Cross‐coupling, Impurities, Palladium, Reproducibility, Transition metals

## Abstract

Over the last decades, numerous efforts have been made to replace metal catalysts with cheaper and earth‐abundant alternatives or to completely exclude metals from cross‐coupling reactions while maintaining high efficiency in the target transformation. However, follow‐up studies often revealed the role of metal impurities in the catalytic process. Thus, active metal impurities can lead to mechanistic misinterpretations, and these could initiate erroneous research directives and lead to severe reproducibility problems. Milestone precedents of the impurity effect in cross‐coupling reactions are well documented in the literature. Interestingly, this fallacy has reoccurred repeatedly over the years due to a lack of thorough mechanistic studies and appropriate research study schemes for identifying impurity effects. Herein, we propose a guideline to elucidate the real catalyst of future catalytic transformations, aiming to reveal the correct mechanism and help exclude the role of impurities in novel catalytic processes. Although this guideline mainly focuses on problems related to trace transition metals, it also offers the basis for more general catalyst research.

## Graphical abstract

Ongoing efforts to discover new catalysts in coupling reactions have provided an effective toolkit for chemists over the last decades. However, metal impurities often lead to costly misinterpretations and reproducibility issues. Our guideline provides a practical roadmap for identifying true catalysts, eliminating impurity‐driven errors, and ensuring reliable research outcomes. Designed primarily for handling trace metal challenges, this framework sets a new standard for robust, impurity‐free catalyst discovery.

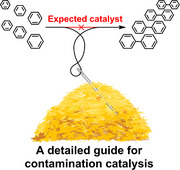

1

Noble metal catalysts are so efficient and versatile that they shape our perception of catalysis. However, they also have drawbacks, including high cost, environmental impact, and, in some cases, adverse health effects. A tremendous amount of research has been devoted to minimizing the noble metal catalyst loading by optimizing reaction conditions and ligands or replacing them with more abundant metals or organic materials. In these latter two research directions, a critical issue must be addressed to establish reproducible catalytic transformations: the “contamination catalysis.” This occurs when an unconsciously added compound is responsible for the transformation.

In a broader context, transition metal impurities can have different impacts on catalysis. Even in minimal amounts, metals can act as both inhibitors and initiators in many fields of chemistry; therefore, their influence on catalytic processes should be considered. In radical polymerizations, the use of metal based initiators is well established,^[^ ^1^ ^]^ and historically, transition metal impurities led to the discovery of efficient Zr‐ and Ti‐based Ziegler–Natta polymerization catalysts.^[^ ^2^, ^3^ ^]^ Among transition metal catalyzed processes in organic synthesis, the Nozaki–Hiyama–Kishi reaction is a notable example of an impurity‐affected transformation. In this process, trace amounts of NiCl_2_ in chromium reagents were found to be the actual catalyst responsible for the transformation. The role of Ni impurities as the active catalyst in cyclopropanation reaction was described by Chen and co‐workers,^[^ ^4^ ^]^ providing an explanation for the irreproducibility of the original “metal‐free” method developed by Franzen and Wittig in the 1960s.^[^ ^5^ ^]^ Impurities can act not only as catalysts but also as inhibitors, as demonstrated by the case of Pb in Simmons–Smith cyclopropanation.^[^ ^6^ ^]^ Many of these historical milestone precedents were excellently summarized and discussed in detail by Thomé, Nijs, and Bolm, along with other catalytic examples (vide infra). Therefore, we do not elaborate on the historical background in such detail.^[^ ^7^ ^]^ Beyond these precedents, there are several examples of the effects of transition metal impurities in the fields of electrochemistry^[^ ^8^, ^9^, ^10^ ^]^ and photocatalysis.^[^ ^11^ ^]^ However, these fields are beyond the primary scope of our scientific perspective and will not be covered extensively. Nevertheless, some aspects of the guideline may still be applicable and useful in these research areas.

Briefly, contamination catalysis plays a dual role in research. In some cases, when identified and managed early in the research process, it can lead to highly beneficial discoveries, such as the development of an efficient industrial catalyst for more economic and sustainable^[^ ^12^ ^]^ catalytic transformations by eliminating inhibitory trace impurities. On the other hand, realizing alternative and improved methods remains a significant challenge. The greater the deviation from the original method, the more critical it becomes to provide experimental and computational mechanistic evidence for the new concept. Additionally, the potential participation of the original metal as a trace contaminant in the novel procedure must be carefully considered to ensure reproducibility and reasonable mechanistic interpretations. In this regard, some practical advice can be found in relevant notes,^[^ ^13^ ^]^ but a more detailed guideline for the analysis and prevention of impurity effects does not exist in the literature. Thus, we aimed to collect the main issues and provide a guide to minimize reproducibility problems and misinterpretations in catalysis.

## 2 Catalyst Reduction, Replacement, and Omission

Among reduction, replacement, and omission, the first option is the easiest to achieve in terms of reproducibility and robustness. In this context, many palladium‐catalyzed methods were efficiently performed with “homeopathic,” ppb–ppm amounts of palladium,^[^ ^14^, ^15^, ^16^ ^]^ for example, with well‐characterized palladium complexes^[^ ^17^, ^18^, ^19^ ^]^ and nanoparticles with various sizes.^[^ ^20^, ^21^, ^22^, ^23^, ^24^ ^]^ In such cases, it is reasonable to assume that the same mechanistic pathways are relevant in the reaction.

Replacement of a known transition metal catalyst with an alternative one is a more challenging task, depending on the reaction type. In this approach, the feasibility of the replacement should be proven, defining the exact role of the new catalyst in the catalytic cycle. Delightfully, there are examples where relatively inexpensive and sustainable metals such as nickel^[^ ^23^, ^25^, ^26^, ^27^, ^28^, ^29^, ^30^ ^]^ and copper^[^ ^31^, ^32^, ^33^, ^34^, ^35^ ^]^ have been shown to act as the catalyst in a broad spectrum of cross‐coupling reactions, replacing more expensive metals.

**Figure 1:**
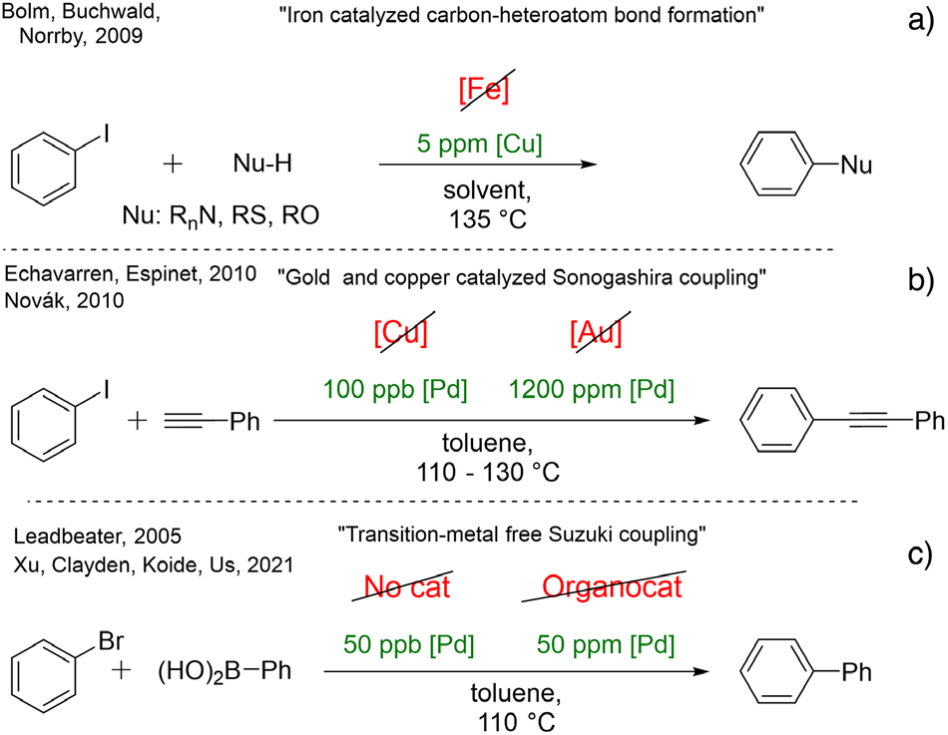
Contamination catalysis in the literature.

However, in certain cases, the impurities in the applied catalyst and additives participate in the reactions. A representative and illustrative example of this effect is the attempted replacement of copper with iron in carbon‐heteroatom bond‐forming coupling reactions.^[^ ^36^, ^37^, ^38^ ^]^ Shortly after the disclosure of these “iron‐catalyzed”‐couplings, Bolm and Buchwald revealed contamination catalysis was caused by ppm levels of copper present in the iron. Consequently, ultra‐pure iron salts did not catalyze the reaction (Figure 1a).^[^ ^39^ ^]^ The beneficial conclusion of these studies is that the couplings can be effectively achieved with ppm levels of copper catalysts.^[^ ^40^ ^]^

Similarly, copper‐^[^ ^41^, ^42^ ^]^ and gold‐catalyzed^[^ ^43^ ^]^ Sonogashira couplings of terminal alkynes and aryl halides are well‐documented in the literature. However, in both cases, ppm/ppb levels of palladium impurities^[^ ^44^ ^]^ were identified as the active catalytic species, which might be called a contamination catalyst (Figure 1b).^[^ ^45^, ^46^ ^]^

Impurities may be present in the catalyst, the base, or even in the solvent or equipment. For instance, in our previous study,^[^ ^46^ ^]^ we showed, in the case of the Sonogashira reaction, that the stir bar previously used in the Pd‐catalyzed reaction could transfer enough palladium into another reaction to reach complete conversion, even after cleaning (not with aqua regia). The stir bar as a source of technical contamination was deeply studied by Janiak^[^ ^47^ ^]^ and Ananikov^[^ ^48^ ^]^ and co‐workers, who made well‐supported scientific statements on the role of metal impurities transported by this common laboratory accessory.

The most rewarding yet synthetically most challenging goal is the complete omission of noble metals from a catalytic process. This approach was also attempted earlier in cross‐coupling reactions,^[^ ^49^ ^]^ but after the first examples of the palladium‐free Suzuki reaction, Leadbeater and co‐workers reassessed their previous results (Figure 1c) and demonstrated that even ppb levels of palladium are able to catalyze the reaction under optimal conditions.^[^ ^50^ ^]^ These findings turned the problem into an advantage: a hyperactive catalytic system and a useful method were discovered for C─C bond formation with the utilization of a minute amount of palladium catalyst.

Due to the potential benefits, these kinds of developments remain the focus of catalytic studies, and researchers are experiencing the same problems, challenges, and difficulties. Recently, an amine‐catalyzed Suzuki–Miyaura‐type coupling reaction was developed (Figure 1c).^[^ ^51^ ^]^ It was demonstrated that specially functionalized aniline derivatives could serve as a catalyst for this reaction. Considering the importance of these findings, the catalysis community was interested in using this new catalyst type, leading to synthetic applications.^[^ ^52^ ^]^ Unfortunately, the palladium contamination that occurred during the preparation of the catalyst was exceptionally difficult to remove using many techniques. As a result, these findings had to be revisited and clarified.^[^ ^53^, ^54^ ^]^

The examples above demonstrate that the identification of contamination catalysis is of utmost importance. This is the most crucial and difficult task, and a healthy suspicion should always be maintained, and warning signs should be examined. Below, we outline the most common indications that should always be inspected both experimentally and computationally to identify potential cases of contamination catalysis.

## 3 The Signs of a Possible Contamination Effect

**Figure 2:**
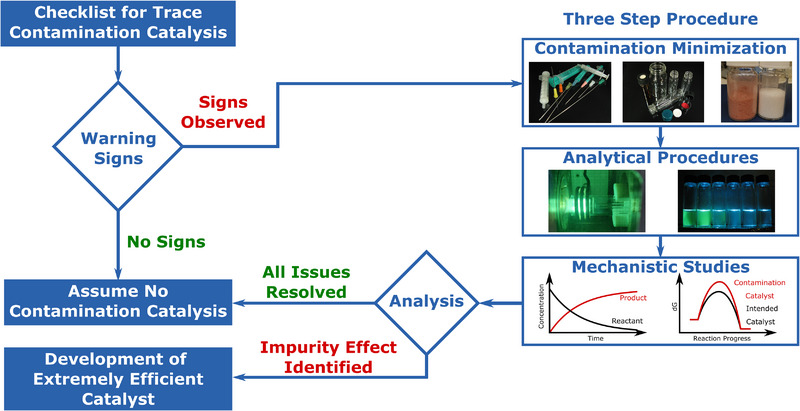
Methods and guidelines for elucidating catalytically active species.

Recognizing when to scrutinize the role of the catalyst is crucial (Figure 2). Based on previous examples, the following phenomena can indicate contamination catalysis.

1. The newly developed reaction exists with the involvement of well‐known catalyst.

*A very similar transformation has already been developed under comparable conditions, using the same starting materials and producing similar products, with utilization of a well‐defined metal catalyst*.

2. Matching reaction scope with literature precedents.

*While investigating the scope, it may become evident that there is a correlation in the reactivity of pairs of substrates (*e.g.*, the effect of electron donating and electron withdrawing groups on the substrates are similar* *)*.

3. High temperature is required for the transformation.

*It seems to be a common observation that at considerably higher temperatures, even trace amounts of catalyst impurities can become effective*.

4. Unexpected reactivity in certain reactions.

*There are certain batches of starting materials that outperform others, or instances where the reaction proceeds much faster or slower than usual. This can indicate the presence of an unintentionally added catalyst. One possible source could be an additive that was added in high excess, including the solvent. Other sources of contamination catalysts include the equipment, particularly the stir bar*.

5. Analogous impurity profile to a known reaction.

*The impurity profile of a reaction is the same or very similar to that of reactions known to be catalyzed by another metal. Side reactions can be a sign of unintentional catalysis*.

6. Theoretical calculation suggests unusually high barrier.

*The computed barriers are not consistent with the experimentally observed reaction rates at the utilized temperatures*.

7. Discrepancies between computational predictions and experimental results.

*Current computational methods are providing robust predictions in terms of the relative trends of activities. Large deviations in trends should not be expected when the correct mechanism is considered*.

If all the above‐mentioned points are negative, one might proceed with the assumption that contamination is not relevant in the given reaction. However, if suspicion remains, a three‐step procedure is recommended to effectively minimize the risk of misinterpretation of catalyst activity caused by contamination catalysis.

1. It is required to establish a laboratory setup and select starting materials that systematically minimize all potential sources of contamination. We provide a detailed description of suitable laboratory practices in the section “Contamination Minimization.”
2. With the usual synthetic setup and minimally contaminated catalytic system in hand, it is essential to assess the overall contamination levels via analytical procedures. We have collected and provided detailed instructions for obtaining reliable results in the section “Analytical Procedures.”
3. Having access to the reaction setups and the corresponding contamination levels, one can study the effect of the contamination on the reaction and its mechanism both experimentally and computationally. To assist with this, we have compiled directly implementable suggestions in the section “Systematic Mechanistic Examinations” to guide readers.

The three‐step procedure described above can aid future methodology developments and serves as a reference for future referees and editors who may have concerns. To support this three‐step procedure, we have provided a detailed “Contamination Catalyst Checklist” with step‐by‐step instructions in the Supporting Information.

## 4 Contamination Minimization

### 4.1 General

A common main problem is equipment and general laboratory purity. Laboratories where transition metals have been extensively used can be a potential source of contamination and should be avoided. Furthermore, the glove box is often a concentrated storage and utilization space of extremely active catalysts of various types; therefore, it should either be avoided or, if not possible, treated with the utmost suspicion.

Residual trace metal impurities on reaction vessels, vacuum lines, and glove boxes can originate from previous reactions and even tap water.^[^ ^11^, ^55^, ^56^ ^]^ Therefore, we generally advise new glassware, especially stir bars, to be used, but equipment can be washed in some cases. Glassware should be fully submerged in freshly prepared *Aqua Regia* overnight, then it should be rinsed with distilled water and submerged in distilled water for 30 min. After rinsing with distilled water, drying in a clean oven is recommended.^[^ ^57^ ^]^ Polyethylene vessels can be purified with nitric acid.^[^ ^58^ ^]^ For vacuum lines, we recommend thorough cleaning by using the method above, along with brand‐new tubes and connectors. As for spatulas and spoons, it is good practice to mark and separate them for each material and purity level, or to use single‐use new plastic ones.

With pure equipment in hand, we must ensure that the main components (by weight) are as pure as possible. These typically include the solvent(s), additives/bases, and starting materials. Solvent(s) should be purified using appropriate methods, such as distillation under an inert atmosphere and with drying agents, depending on their properties and required purity. Commercially available, highest‐purity solvents can be used without further purification. For additives and bases, acceptable purity can be ensured by sourcing the available highest purity from reliable suppliers and storing them under proper conditions to prevent contamination. Starting materials should be purified using techniques such as recrystallization, column chromatography, or sublimation, with purity confirmed through high‐performance liquid chromatography (HPLC) or mass spectrometry (MS). If necessary, inductively coupled plasma mass spectrometry (ICP‐MS) can be used to detect trace elements beyond the expected components of the given compound. For a more comprehensive description of purification techniques and best practices, we recommend using available literature on purification,^[^ ^59^ ^]^ which provides detailed methods for ensuring high‐purity reagents and materials.

In general, different metals are often found together in the production process of inorganic chemicals due to their natural abundance. Therefore, simple metal salts with acceptable purity for organic chemistry (>95%) are not appropriate for catalytic studies. Any possible contamination should be minimized for these critical reaction components, and the metal source should be of the highest purity available (>99.99%). It should be noted that a material with a purity of 99.999% still contains 10 µg g^−1^ impurities. Depending on the reaction setup, this alone can introduce 10 ppm of an unknown potential catalyst, which can divert the reaction route in many cases. The use of mass percent (w/w %) as a measure of purity can be misleading, as materials with high molecular weight can contain significantly larger amounts of impurity. For example, cesium carbonate is very useful due to its relatively good solubility in organic solvents. However, it contains more than three times the amount of impurities compared to its sodium counterpart with the same w/w %.

Even with efforts made to ensure equipment and material purity, contamination cannot be ruled out, and it is insufficient to compare the new conditions to existing protocols. A blank reaction without the catalyst candidate should be conducted to confirm that the reaction does not proceed without the intended catalyst (background reaction/catalyst).

Especially for such challenging reactions, reproducibility must be ensured.^[^ ^60^, ^61^ ^]^ First, batch‐to‐batch reproducibility should be confirmed (using a new batch of starting materials for catalyst, substrates, and solvent from different suppliers). Subsequently, robustness should be validated under as independent conditions as possible, preferably in an independent research group. Ideally, the results should be replicated using the least synthetically advanced, locally sourced starting materials in another laboratory.

However, it should be kept in mind that “contamination catalysis” cannot be completely excluded even in this scenario.

### 4.2 Purity of “Surrogate Metal Catalyst”

If a well‐established chemical transformation requires a very expensive and/or toxic metal‐based catalyst, it might be beneficial to use a cheaper or otherwise better catalyst, so called the “surrogate metal catalyst.” However, these metals often come from the same group of metals, assuming that their chemical similarity can be exploited through optimizing a better catalyst.

Several transition metals co‐occur in nature and may even be extracted from the same ore. In‐process purifications can be difficult due to the chemical similarities between these metals. This issue can be even worse: when the natural occurrence of a similar metal is not high enough for the metal manufacturer to extract and remove it, then these metals are present as an impurity. This fact could lead to the introduction of unexpected metal contaminants into the reaction mixtures by the main metal catalyst. Unfortunately, comprehensive data on typical metal contaminations of the elements is unavailable for users; we have collected some sources to represent the complexity of this issue. For example, most platinum group metals are derived from nickel mining. These metals are extracted together, and the initial steps in the purification process to obtain platinum metals involve the removal of nickel and then copper. Subsequently, through mostly solution refining, silver, gold, palladium, ruthenium, osmium, platinum, iridium, and rhodium are separated in this order.^[^ ^62^ ^]^ Similarly to the co‐extracted platinum metals, cobalt is also extracted from copper and nickel ore.^[^ ^63^ ^]^ High‐purity gold may contain Pd, Fe, Cu, and Ag in the 10 µg g^−1^ range. The exact amount is highly variable due to macroscopic inhomogeneities resulting from different melting points.^[^ ^64^, ^65^ ^]^ Other pure metals (Hg, Ga, In, Sn, Zn, Al, Ag, Au, Cu) used as standards for temperature scales can contain several impurities.^[^ ^66^ ^]^ Iron may contain copper, lead and zinc, calcium, aluminum, and manganese. ^[^ ^39^, ^67^, ^68^ ^]^ Lithium carbonate (98+%) may also contain copper, iron, and nickel, in addition to the expected alkali and alkali‐earth metals, at concentrations of hundreds of mg kg^−1^.^[^ ^69^ ^]^

In this context, we can always reasonably expect cross‐contamination among these metals, and to avoid the involvement of a “surrogate metal catalyst,” it is strongly advised to check the purity data sheet of suppliers, and if it is necessary, analyze the minor components of the metal catalyst by ICP‐MS. With this, we cannot rule out all unwanted effects, but we can consider other catalytic scenarios and identify critical points of the reaction mechanism.

As an additional note regarding noble metals, we should consider that platinum and palladium metals are part of everyday life through automotive catalysts, and concentrations can exceed 100 µg kg^−1^ in road dust on some busy roads.^[^ ^70^ ^]^ Even potatoes can contain µg kg^−1^ quantities of platinum and ng kg^−1^ quantities of rhodium.^[^ ^71^ ^]^ Thus, not only the commercial chemicals can contaminate our reaction, but we can introduce trace metals from many sources if materials are not appropriately handled.

### 4.3 Purity of Potential Organocatalyst

Developing a good organocatalyst is highly beneficial for many applications, especially in industrial (pharmaceutical) setups. However, in the preparation of organocatalysts, it is crucial to avoid the usage of any transition metal catalysts, particularly those that could be applied as catalysts in the target reaction. Furthermore, the source of chemicals should be carefully selected, as traces of metals are often carried forward through several steps in a multi‐step synthesis. Once synthesized, the purification of the catalyst candidate is an essential but challenging task.

Chromatographic techniques are a well‐known and useful method for the purification of organic chemicals. On the other hand, on their own, they are not recommended as the sole method for purification. In cases where a very minor component might be present, there is a high risk of co‐elution, as interactions with the desired organic compound can hinder the separation process.

On the other hand, careful recrystallization(s), where medium‐size crystals are formed, is an efficient technique to reach sufficient catalyst purity. Distillation and sublimation might also work, but they are usually not a practical solution below a certain sample mass.

In addition to general purification techniques, transition metal scavengers can be applied to reduce the content of these elements. However, this method has limitations that must be considered. Namely, these processes have to be carefully optimized for the given application, and even then, residual metal concentrations in the ppm range may still remain.^[^ ^72^ ^]^ This persistence is due to two remaining obstacles. First, if the organic compound has the ability to form even weak complexes with the metal, it will likely exist in the complex form, given that the concentration of the organic compound is orders of magnitude higher. Second, the mixture might contain a very stable pre‐formed complex, which is only minimally affected by the scavenger.

It is also possible to purify organic materials through electrochemical methods by depositing them on an electrode. This approach can also be useful in qualitative analysis, as the deposited metal concentrates on the electrode and can be analyzed.^[^ ^73^ ^]^

If the organic catalyst is a polymeric material, its proper purification is an especially difficult task. One possible approach is to use GPC. Using this technique for the purification of a suspected polymeric photocatalyst, Kosco and co‐workers successfully reduced the Pd level of their polymer, synthesized by Suzuki coupling, from 1170 ppm to below their detection limit of 1 ppm.^[^ ^74^ ^]^

Molecules with more polar functional groups are more susceptible to accumulating metal impurities. Consequently, pharmaceuticals and amino acids are prone to high amounts of metal contamination, including iron, nickel, copper, and chromium.^[^ ^75^, ^76^, ^77^, ^78^ ^]^

## 5 Analytical Procedures

ICP is the main tool for the identification of possible transition metal contaminants, but sample preparation can be overseen if the samples are not prepared carefully. Below, we provide our recommendation for proper sample preparation.

Adequately cleaned dishes and high‐purity acids are essential for proper sample preparation. The digestion vessels must be pre‐washed with ultrapure water (18.2 MΩ cm^−1^) using dilute acids (nitric acid and/or hydrochloric acid) for an acidic wash.

Samples should undergo complete digestion with microwave‐assisted acidic treatment.^[^ ^79^ ^]^ For this, high‐purity concentrated nitric acid and hydrochloric acid must be used in a ratio of inverse aqua regia (cc. HNO_3_ and cc. HCl 3:1). Nitric acid oxidizes the organic components of the sample, while hydrochloric acid ensures that transition metals remain in solution.

The solutions obtained by microwave digestion must be brought to final volume with ultrapure water (18.2 MΩ cm^−1^). The resulting solutions should be analyzed with an ICP‐OES or ICP‐MS instrument. Depending on the metal concentration, one or both techniques have to be used.

External calibration should be performed using single‐ or multi‐element ICP standard solutions, and a three‐to‐four‐point calibration curve should be measured to calculate the metal content of the samples. If the sample amount allows, it is advisable to perform two or three parallel ICP measurements with three independent sample preparations.

For concentrations near the detection limit, a spiking experiment should be conducted, and spike recovery should be reported.

Another useful tool in the search for transition metals is the utilization of fluorescent sensors. Quantitative measurements at the ppm level can be achieved for certain metals using relatively simple sensors and equipment. However, a drawback could be that some sensors are specific to certain elements, so one has to know what to look for. There are several methods for the detection of transition metals via fluorescence.^[^ ^80^, ^81^, ^82^, ^83^ ^]^ A more organic chemistry‐focused application for palladium has been developed by the Koide research group.^[^ ^84^, ^85^, ^86^, ^87^ ^]^

While NMR, as used by synthetic chemists, is not particularly effective in the identification of very minor unknown impurities, it might give some clues.^[^ ^73^ ^]^ Pay attention to minor peaks and attempt to trace back their origin. Run NMR for nuclei that are not apparently present, such as phosphorus, nitrogen, or fluorine. In some cases, strongly binding ligands might be present and visible to a keen observer.

### 5.1 Systematic Mechanistic Examinations

One possible approach to determine whether (transition) metal contamination plays a substantial role in a given transformation is to systematically study its reaction mechanism. The most often used methods for this purpose are experimental or computational kinetics, as well as estimates of isotope and substituent effects.

Conceptually, both the contamination‐free and spiked reactions must be studied to exclude the catalytic activity of the contaminant. Technically, a completely contamination‐free system is experimentally never guaranteed, although purity can be pursued (see Section 3).

To rule out the possibility that the reaction is driven by impurities, rigorous kinetic experiments would be extremely valuable but tedious. Fortunately, a lot of information can be gathered from simplified experiments. For a quick estimate, one might choose a certain point at intermediate conversion (i.e., 70% yield) for comparison. Monitoring the reaction over time and comparing time‐yield curves can reveal mechanistic similarities. While one reaction might proceed faster, the shape of the curve might provide valuable insight.

The above‐mentioned spiking experiments should be conducted carefully, as there is often‐limited information on the actual catalyst amount. If there is already too much catalyst present from the reaction matrix, adding too little of the suspected contaminant may not affect the reaction outcome. A contamination catalyst can be active on the ppm or even the ppb scale. Therefore, spiking a blank reaction with various amounts of suspicious metal from ppb to low mol% should be examined. Even with this careful approach, it is important to remember that a synergistic effect may complicate the reaction through the interaction between two metals or between metal contamination and an organic substance having chelating properties.

### 5.2 Computational Aspects

In contrast to experimental setups, computational approaches provide access to both typical metal‐catalyzed pathways and proposed alternative mechanisms. Therefore, it should be proven that the suggested catalyst is substantially more effective than the contaminant at the maximal possible experimental contamination level. This procedure is subject to some of the usual tricks of the trade,^[^ ^88^ ^]^ such as the general requirement for conformational analysis,^[^ ^89^ ^]^ proper concentration corrections, including implicit and, if necessary, explicit solvation, and the identification of resting states and rate‐determining steps for both mechanisms.^[^ ^90^, ^91^ ^]^

However, some special issues arise due to the nature of the problem. First, because the contaminant is present at an extremely low concentration in one mechanism but completely absent in the other, barrier heights can only be directly compared if the dopant concentration is explicitly taken into account instead of using the usual 1 mol dm^−3^ concentration. Second, because insoluble reactants (i.e., solid alkaline compounds) are often used, special attention has to be paid to their handling. Ideally, one should consider computer simulation of the solid‐liquid interfaces via *ab initio* molecular dynamics simulations (AIMD). As an alternative approach, cluster‐continuum calculations have been suggested and complemented by AIMD determination of the solution phase structure.^[^ ^92^ ^]^ Despite the theoretical rigor of these approaches, computational studies involving these techniques are rarely feasible. Therefore, more pragmatic but approximate solutions have been suggested to estimate the Gibbs free energy of these solid additives on a consistent scale with the quantum chemical calculations. One option is to combine thermodynamic cycles with experimental and computational data.^[^ ^93^ ^]^ Although this approach is very straightforward, it is prone to sizeable errors due to species with highly different electronic structures.^[^ ^94^ ^]^ Further options might include purely computational approaches to thermodynamic cycles, including solid state, gas phase, and implicit solvent calculations^[^ ^93^ ^]^ or extrapolations to provide a lower boundary to the solvation free energies via increasing size cluster models,^[^ ^95^, ^96^ ^]^ Ultimately, the replacement of the solid reactants should be experimentally attempted to obtain a model reaction that can be followed via homogeneous phase spectroscopy and computational chemistry as well.

Given the uncertainty arising from the above‐mentioned challenges, we suggest studying substituent effects or kinetic isotope effects if applicable. Substituent effects might provide different trends for the two mechanisms. Therefore, if an existing transformation with another catalyst is similar to the newly developed method, it is highly recommended to compare the two reactions. To this goal, multiple compounds from the substrate scope of the other reaction with versatile substitution patterns should be selected. Then, the kinetics and conversions of both reactions should be compared, along with their corresponding computational estimates. NMR or GC measurements from the crude reaction mixtures are suggested to avoid added errors by work‐up techniques.

Even in the absence of well‐defined contamination‐free or metal‐catalyzed analogous reactions, we suggest considering quantities depending on relative rates since trends are more robust with respect to technical issues than absolute rate constants. The same applies to kinetic isotope effect measurements and calculations, which benefit considerably from error cancellation.

## 6 Conclusion

We have composed a guideline as a recommendation for future methodology developments, emphasizing the importance of proper handling of materials and equipment, purification, and analysis of products to prevent any mechanistic misinterpretations. We are aware that complying with all of our recommendations is a demanding endeavor, but adhering to the presented guidelines and reporting the corresponding results will highly increase the credibility of newly suggested synthetic procedures. By addressing these critical issues, researchers can save valuable resources by identifying and addressing contamination‐related problems early in their studies. Ultimately, we hope that this approach could transform the curse of any impurity effect into the blessing of identifying novel and highly efficient catalyst systems in chemical research.^[^ ^97^ ^]^

## Conflict of Interests

The authors declare no conflict of interest.

## Supporting information



Supporting Information

Contamination Catalysis Checklist

## Data Availability

The data that support the findings of this study are available in the supplementary material of this article.
