# Supplementary material for: Guideline for Analysis and Prevention of Contamination Catalysis
Source: Angew Chem Int Ed Engl. 2025 Apr 30;64(26):e202424425. doi: 10.1002/anie.202424425 (PMC12184295; doi:10.1002/anie.202424425)
Supplement: Supplementary file 1 — Supporting Information [file ANIE-64-e202424425-s001.pdf]

Contamination Catalysis Checklist

Supplementary material for: Guideline for Analysis and Prevention of Contamination Catalysis

János Daru,[a] Zsombor Gonda,[b] Zoltán May,[c] Zoltán Novák,*[d] Gergely L. Tolnai*[b]*
**Email: janos.daru@ttk.elte.hu, novakz@ttk.elte.hu, tolnai@chem.elte.hu*

Details of current research

Title:

Dramatic Impact of ppb Levels of Palladium on the "Copper-Catalyzed" Sonogashira Coupling

Authors:

Zsombor Gonda, Gergely L. Tolnai, Zoltán Novák

Affiliation:

Eötvös University, Institute of Chemistry, Pázmány Péter stny 1/A, 1117 Budapest (Hungary),

I. Contamination Minimization

I.1 General

- ? **Vacuum line cleaned**
- ✓ **Did not use glovebox**
- ? **New equipment was used (glassware, spatula, syringes, needles, Hamilton)**
- ? **Glassware washed in aqua regia**
- Solvent
  - ? **Solvent distilled**
  - ? **Used HPLC solvent**
- ✓ **None of the starting materials are made via transition metal catalyzed reaction**
- ? **Blank reaction with no added catalyst provided no yield**
- ? **No unexplained repodicibility events happened during experimentation**

IN THIS SECTION A REPRESENTATIVE REACTION MIXTURE SHOULD BE DESCRIBED. **ADD SOLVENTS FIRST,** THEN EVERY OTHER COMPOUND

- ? **Added substances are as pure as it can get**

Scale of reaction:

Starting material: 1 mmol

Mass of whole reaction mixture including reactants and solvents: 2800 mg

Substance name: toluene

Purity(%): 99.8

Added mass (mg): 1734 There is 3.4680 mg of impurity in toluene.

Substance name: Cu(PPh3)2NO2

Purity(%): 98

Added mass (mg): 163 There is 3.2600 mg of impurity in Cu(PPh3)2NO2.

Substance name: K2CO3

Purity(%): 98

Added mass (mg): 276 There is 5.5200 mg of impurity in K2CO3.

Substance name: hexylamine

Purity(%): 98

Added mass (mg): 28 There is 0.5600 mg of impurity in hexylamine.

Substance name: iodobenzene

Purity(%): 98

Added mass (mg): 204 There is 4.0800 mg of impurity in iodobenzene.

Substance name: phenylacetylene

Purity(%): 98

Added mass (mg): 102 There is 2.0400 mg of impurity in phenylacetylene.

Total Impurity Content: 18.9280 mg. That is in total 0.0068 mg/mg impurity.

- ☑ Reaction is verified by independent source
  - Personal
    - ☑ Another group member
    - ☐ Someone from the same faculty
    - ☐ From independent institution
  - Technical
    - ☐ The catalyst was prepared independently
    - [?] Additives and other materials are sourced independently

yield by GC	Source 1	Suorce 2	Source 3
Additive 1 CuI(PPh)3I	(Aldrich 98%) 99.7 %	(Aldrich 99.999%) 17 %	
Additive 2 Cs2CO3	(unknown, 98%) 81.3 %	(AlfaAesar 99.999%) 15%	

I. 2 Surrogate Metal Catalyst

- [?] The new metal does not come from the same mining process as an existing catalyst
-

- [ ] Catalyst is purifed by
  - [ ] Column chromatography
  - [x] Recrystallization
- [x] Ligand(s) are not prepared by metal coupling reaction
- [?] Ligand is purifed by
  - [ ] Column chromatography
  - [ ] Recrystallization

I. 3 Organic Catalyst

- [ ] The catalyst is not prepared by metal coupling reaction
- [ ] The starting materials for the catalyst are not prepared by metal catalyzed reactions
- [ ] Metal scavengers are used
  - Conditions: ______
- [ ] Catalyst is purifed by
  - [ ] Column chromatography
  - [ ] Recrystallization
  - [ ] Distillation
  - [ ] Electrodepositing
  - [ ] GPC
  - [ ] Sublimation
  - [ ] Gel electrophoresis

II. Analytics

- [ ] ICP
  - ICP Equipment: ______
  - Confirmed detection limit: ______
  - Sample preparation details: ______
  - Spike and spike recovery: ______
  - Results:
    - Catalyst: ______
    - Whole reaction mixture: ______
- [ ] Fluorometric analysis details: ______
- [ ] All minor impurity peaks are assigned in NMR, notes: ______
- [ ] No unknown GC peaks are observed, note: ______
- [ ] No unknown HPLC peaks are observed, note: ______
- [ ] Other: ______

III. Systematic mechanistic examinations

- [?] Impurity profile is different
  - [ ] Quantitatively

- [?] Qualitatively

- [ ] The reaction time-yield curve is different.
- [ ] The chosen starting materials yield against the existing reaction is different.

**yield**	**Substrate 1**	**Substrate 2**	**Substrate 3**	**Substrate 4**	**Substrate 5**
Reaction 1					
Reaction 2					

- [ ] Spiking
- [ ] Spiking of blank
  - Obtained yield and conversion:
- [ ] Different batches of catalyst performs adequately similar by (NMR, GC) conversion. Method:

  Yields (%):
  - Batch 1:
  - Batch 2:
  - Batch 3:

Please add yields from reactions with the same compound with different batches of catalyst.

- [ ] Height of the rate-determining activation barrier in the absence of contaminant:
  - Calculated barrier
- [ ] Height of the rate-determining activation barrier assuming contaminant catalysis
  - Calculated barrier
- [?] Temperature of the reaction
  - T(°C) 110 °C
- [ ] Solid species were considered for Gibbs free energy calculations.
  - Method:
- [ ] The predicted contamination-free activation free energies and the measured reaction rates are correlated
  - Rates and difference:

Predicted rate	Measured rate	Difference

- [ ] Measured kinetic isotope effect:
- [ ] Calculated kinetic isotope effect:

Checked: 6 | Needs further assessment: 14

Additional Comments

This is a report on our "palladium free" sonogashira coupling, prior really noticing, that Pd was the real catalyst in our case. The most awareness raising features are: Irreproducibility, especially upon changing source of additive, and high temperature required. Upon further investigations, the same impurity profile(homocoupled products), the spiking experiments and the ICP analysis proved the presence of Pd. To conclude the real catalyst, we ran kinetic experiments.
