# Supplementary material for: Guideline for Analysis and Prevention of Contamination Catalysis
Source: Angew Chem Int Ed Engl. 2025 Apr 30;64(26):e202424425. doi: 10.1002/anie.202424425 (PMC12184295; doi:10.1002/anie.202424425)
Supplement: Supplementary file 2 — Contamination Catalysis Checklist [file ANIE-64-e202424425-s002.html]

Contamination Catalysis Checklist


×

To be able to reload inputs, the Checklist uses locally stored cookies.

# Contamination Catalysis Checklist

*### Supplementary material for: Guideline for Analysis and Prevention of Contamination Catalysis

  
  
János Daru,\*[a] Zsombor Gonda,[b] Zoltán May,[c] Zoltán Novák,\*[d] Gergely L. Tolnai\*[b]  

[a] Department of Organic Chemistry, Eötvös Loránd University, Institute of Chemistry, Pázmány Péter stny 1/a, H1117 Budapest, Hungary  
[b] ELTE Novel Scaffolds Research Group, Eötvös Loránd University, Institute of Chemistry, Pázmány Péter stny. 1/A, H-1117 Budapest, Hungary  
[c] Research Centre for Natural Sciences, HUN-REN, Magyar Tudósok körútja 2, H-1117 Budapest, Hungary  
[d] MTA-ELTE “Lendület” Catalysis and Organic Synthesis Research Group, Eötvös Loránd University, Institute of Chemistry, Pázmány Péter stny. 1/A, H-1117 Budapest, Hungary

\*Email: janos.daru@ttk.elte.hu, novakz@ttk.elte.hu, tolnai@chem.elte.hu*  
  
 Users manual 

## Details of current research

#### *Title:*

### 

Enter

#### *Authors:*

### 

Enter

#### *Affiliation:*

##### 

Enter

## I. Contamination Minimization

### I.1 General

- **Vacuum line cleaned**

  *(!)*
  Very important entry
- **Did not use glovebox**

  *(!)*
  Very important entry
- **New equipment was used (glassware, spatula, syringes, needles, Hamilton)** 

  *(!)*
  Very important entry
- **Glassware washed in aqua regia**

  *(!)*
  Very important entry
- Solvent
  - **Solvent distilled**

    *(!)*
    Very important entry
- **Used HPLC solvent**

  *(!)*
  Very important entry

- **None of the starting materials are made via transition metal catalyzed reaction**

  *(!)*
  Very important entry
- **Blank reaction with no added catalyst provided no yield**

  *(!)*
  Very important entry
- **No unexplained repoducibility events happened during experimentation**

  *(!)*
  Very important entry
  
 IN THIS SECTION A REPRESENTATIVE REACTION MIXTURE SHOULD BE DESCRIBED. **ADD SOLVENTS FIRST,** THEN EVERY OTHER COMPOUND

*info*
This will calculate the total impurity content and might give hints where to look for. NMR-pure compounds are generrally assumed to be 95% pure. The "added compounds" will not reload from cookies.

  
  
- **Added substances are as pure as it can get** 

  *(!)*
  Very important entry
Scale of reaction:  

Starting material: mmol  
Mass of whole reaction mixture including reactants and solvents:  mg
  

|  |  |
| --- | --- |
| Substance name: |  |
| Purity(%): |  |
| Added mass (mg): |  |
|

Add Compound
  
- **Reaction is verified by independent source**

  *(!)*
  Very important entry

- Personal

- Another group member
- Someone from the same faculty
- From independent institution

- Technical

- The catalyst was prepared independently
- Additives and other materials are sourced independently

  | yield | Source 1 | Suorce 2 | Source 3 |
  | --- | --- | --- | --- |
  | Additive 1 |  |  |  |
  | Additive 2 |  |  |  |

### I. 2 Surrogate Metal Catalyst

- The new metal does not come from the same mining process as an existing catalyst

  *info*
  See the full text for clues, and ask the provider
- Catalyst is purifed by

- Column chromatography
- Recrystallization

- Ligand(s) are not prepared by metal coupling reaction
- Ligand is purifed by

- Column chromatography
- Recrystallization

### I. 3 Organic Catalyst

- The catalyst is not prepared by metal coupling reaction
- The starting materials for the catalyst are not prepared by metal catalyzed reactions
- Metal scavengers are used

|  |  |
| --- | --- |
| - Conditions: |  |


- Catalyst is purifed by

- Column chromatography
- Recrystallization
- Distillation
- Electrodepositing
- GPC
- Sublimation
- Gel electrophoresis

## II. Analytics

- **ICP**

  *(!)*
  Very important entry

|  |  |
| --- | --- |
| - ICP Equipment: |  |
| - Confirmed detection limit: |  |
| - Sample preparation details: |  |
| - Spike and spike recovery: |  |

- Results:

- Catalyst: |  |

- Whole reaction mixture: |  |


- Fluorometric analysis details:
- All minor impurity peaks are assigned in NMR, notes:
- No unknown GC peaks are observed, note:
- No unknown HPLC peaks are observed, note:
- Other:

## III. Systematic mechanistic examinations

- Impurity profile is different

  *info*
  The side products and/or impurities are different in the reaction in question, then literature precendences

- Quantitatively
- Qualitatively

- The reaction time-yield curve is different.

  *info*
  This refers more to the shape of the curve then the speed
- The chosen starting materials yield against the existing reaction is different.

  *info*
  If there is a reaction with similar scope profile choose 5 substrates to compare with the new reaction.

| yield | Substrate 1 | Substrate 2 | Substrate 3 | Substrate 4 | Substrate 5 |
| --- | --- | --- | --- | --- | --- |
| Reaction 1 |  |  |  |  |  |
| Reaction 2 |  |  |  |  |  |

- Spiking
- Spiking of blank
- - Obtained yield and conversion: |  |
- Different batches of catalyst performs adequately similar by (NMR, GC) conversion. Method:

Yields (%):- Batch 1:
- Batch 2:
- Batch 3:

  
- Height of the rate-determining activation barrier in the absence of contaminant:

  - Calculated barrier |  |
- Height of the rate-determining activation barrier assuming contaminant catalysis

  - Calculated barrier |  |
- **Temperature of the reaction**

  *(!)*
  Very important entry

  - T(°C) |  |
- Solid species were considered for Gibbs free energy calculations.

  - Method: |  |
- The predicted contamination-free activation free energies and the measured reaction rates are correlated

  - Rates and difference:

  |  |  |  |
  | --- | --- | --- |
  | Predicted rate | Measured rate | Difference |
  |  |  |  |- Measured kinetic isotope effect:

    |  |
  - Calculated kinetic isotope effect:

    |  |

  
**Checked: 0 | Needs furter assessment: 0**

### Additional Comments

### Print page to PDF, then save

  

Print Page
